# Ethical and Scientific Pitfalls Concerning Laboratory Research with Non-Human Primates, and Possible Solutions

**DOI:** 10.3390/ani9010012

**Published:** 2018-12-29

**Authors:** Constança Carvalho, Augusta Gaspar, Andrew Knight, Luís Vicente

**Affiliations:** 1 Centre for Philosophy of Science of the University of Lisbon, Department Animal Biology, Faculty of Sciences, University of Lisbon, Lisbon 1749-016, Portugal; lmvicente@fc.ul.pt; 2 Catolica Research Center for Psychological, Family and Social Wellbeing (CRC-W), Universidade Católica Portuguesa, Palma de Cima, Lisboa 1649-023, Portugal; augusta.gaspar@fch.lisboa.ucp.pt; 3 Centre for Animal Welfare, Faculty of Humanities and Social Sciences, University of Winchester, Winchester SO22 4NR, UK; Andrew.Knight@winchester.ac.uk

**Keywords:** non-human primate research, biomedical research, deontology, utilitarianism, animal use alternatives

## Abstract

**Simple Summary:**

Legislation and guidelines governing biomedical research with humans and non-human primates (NHPs) rely on different ethical frameworks. In this paper we argue that the main ethical framework used to assess and justify NHP experimentation is inadequate for its purpose. We propose a change of framework that we believe would benefit NHPs and improve research quality.

**Abstract:**

Basic and applied laboratory research, whenever intrusive or invasive, presents substantial ethical challenges for ethical committees, be it with human beings or with non-human animals. In this paper we discuss the use of non-human primates (NHPs), mostly as animal models, in laboratory based research. We examine the two ethical frameworks that support current legislation and guidelines: deontology and utilitarianism. While human based research is regulated under deontological principles, guidelines for laboratory animal research rely on utilitarianism. We argue that the utilitarian framework is inadequate for this purpose: on the one hand, it is almost impossible to accurately predict the benefits of a study for all potential stakeholders; and on the other hand, harm inflicted on NHPs (and other animals) used in laboratory research is extensive despite the increasing efforts of ethics committees and the research community to address this. Although deontology and utilitarianism are both valid ethical frameworks, we advocate that a deontological approach is more suitable, since we arguably have moral duties to NHPs. We provide suggestions on how to ensure that research currently conducted in laboratory settings shifts towards approaches that abide by deontological principles. We assert that this would not impede reasonable scientific research.

## 1. Non-Human Primates in Laboratory Research

Since the mid twentieth century, non-human primates (NHPs) have been widely used in laboratory research, mostly in biomedical research [1], and mostly in the cognitive sciences [2].

In recent years, due to public pressure and legislation, the number of NHPs used in biomedical research has been significantly reduced in the European Union and the United States [3,4], but has increased dramatically in some other countries, particularly China [5].

Some researchers claim that the use of NHPs in biomedical research is crucial, due to their similarities with humans [6,7], and state that a total ban on such research would compromise medical advances in several fields, such as those focused on infectious diseases, cardiovascular diseases, endocrine diseases, reproductive diseases, neurological disorders, ophthalmic diseases [5,6] asthma, certain types of cancer [1], transplants [8] and psychiatric disorders [4]. However, for many years the presumed benefits of NHP research for medical advances were not subjected to rigorous critical evaluation. In recent years however, evidence-based assessments have been conducted, frequently demonstrating that NHP models have provided disappointing contributions toward human medical advancements [9,10,11,12]. 

In the cognition and behaviour domains, studies with captive primates have made relevant contributions to psychology and neuroscience, as exemplified by Harlow’s experiments on “the nature of love” [13] or Selemon and Goldman Rakic’s [14] early brain topography studies. In both cases, as in many others, discoveries were made which incurred high costs to NHP subjects, including suffering and death. The ecological validity of behavioural and cognitive studies conducted on captive NHPs has also been questioned [15]. 

## 2. Similarities between Humans and Non-Human Primates

Moral concerns raised by the use of NHPs in intrusive or invasive research result from their sentience, consciousness and affective states. In those aspects, NHPs are very similar to humans, which makes it reasonable to give them similar protection to that afforded to human subjects. However, they are very different in other aspects, so they are not necessarily good models for human biology.

From the mid-twentieth century onwards, it became clear that NHPs and humans shared so many traits that trying to categorise any trait as wholly human became something of a futile exercise. Below, we present a few examples of studies that brought a greater awareness of the high similarity (and evolutionary continuity) between humans and NHPs. 

Some of the very first “humanlike” capabilities that attracted considerable scientific interest were the discoveries that NHPs build and use tools [16,17], solve new problems, and develop and pass on cultural behaviour [18,19]. NHPs of several species have also demonstrated the ability to recognise themselves in a mirror―an ability that has been largely interpreted as evidence of self-awareness [20,21]. This was once thought to be a uniquely human ability. All NHPs that have been studied to date, from rhesus monkeys to chimpanzees and gorillas, have also been shown to have distinct personalities with complex behavioural patterns, as occurs with humans [22]. Furthermore, all NHPs establish strong social bonds [23,24], and most live in complex societies [25]. Like humans, NHPs experience and display emotions [26], strong mother–infant and other familial bonds [27] and are capable of experiencing empathy and behaving sympathetically (e.g., [28,29]). In addition, an increasing amount of evidence has accumulated that they have notions of justice and unfairness [30]. NHPs communicate effectively through vocalisations, gestures, and facial expressions [31,32,33,34,35]. They possess a linguistic and lateralised brain which allows them to learn and use sign language, among other skills [36,37]. NHP cognitive abilities have been astounding us for many years. NHPs can create lasting memories [38,39], possess mathematical skills [40], and can even outperform university students in numerical memory [41]. They can solve complex problems that require intelligence [42,43,44].

These skills are not exclusive to NHPs and can be found across a number of other non-human animal species. However, NHP behaviour and skills are among the most well documented. The fact that they are our closest living relatives has probably facilitated the recognition and social acceptance of their cognitive abilities and emotional lives and has probably made them a preferred target for cognitive research. 

Collectively, these and many other studies addressing primate cognition, emotion, and social behaviour have become the scientific basis for arguing that NHPs should be afforded a significant moral status, for some authors [45,46]. It has also been pointed out that the similarities between humans and NHPs are the main ethical obstacle regarding the laboratory confinement and use of NHPs [47]. This is indeed a controversial issue within the scientific community, and for the wider public [48,49], but the recognition that there are significant ethical concerns to be addressed is nearly universal.

Because of their anatomical and physiological similarity to humans [50], as well as such cognitive, behavioural, and social similarities, NHPs have been portrayed as ideal animal models for some biomedical and cognitive research problems. 

However, such similarities do not automatically make NHPs ideal models for humans within biomedical research [15]. For example, major evolutionary jumps have occurred since the last common ancestor humans shared with chimpanzees, with homologous brain areas being recruited in humans for new functions, and new structures emerging altogether [51].

## 3. The Ethical Frameworks of Deontology and Utilitarianism 

Biomedical research, with both humans and non-human animals, presents considerable ethical challenges, since it is not uncommonly invasive or intrusive, causing pain, stress or discomfort. For example, xenotransplantation experiments are classified under the current legislation as “severe” procedures, since they are likely to compromise the general health of the animal in the case of organ rejection. However, NHPs are still presently used in this sort of research [52,53].

In modern human societies, laws should express and enforce society’s moral codes [54]. Legislation and ethical guidelines have arisen to guide scientists through ethical dilemmas and prevent forms of abuse that were more common in the past. Classical examples include the use of orphans to carry smallpox live vaccine through arm-to-arm transportation across the Atlantic Ocean during the 19th century―this involves vaccinating a child and then transferring the vaccine to another as soon as the infectious pustule forms [55]; medical research conducted with prisoners by German doctors; and the infamous Tuskegee research, in which African-Americans that had syphilis unknowingly were not given treatment so the doctors could study the natural progress of the disease in rural American areas between 1932 and 1972 [56]. After World War II and the subsequent Nuremberg trials, rules and principles to guide research with human beings emerged. The general rules that guide modern research with human subjects were written by the Council for International Organizations of Medical Sciences (CIOMS) in collaboration with the World Health Organization (WHO) in 1982 and revised in 1993 and 2002 [57]. The International Ethical Guidelines for Biomedical Research Involving Human Subjects―which has been transposed into legislation or guidelines in most countries, established four basic ethical principles: respect for persons, beneficence, non-maleficence and justice. 

Respect for persons includes the principle of autonomy (as described by Beauchamp and Childress [58]), and the protection of individuals with impaired autonomy [57]. The beneficence principle refers to the obligation to maximise benefit, whilst the non-maleficence principle refers to minimising harm [57,58], in keeping with the utilitarianism view (see section below), except that in this case the permissible harm must be mild, regardless of expected benefits [58,59]. The principle of justice requires the equitable distribution of resources, which in the case of biomedical research translates to an equal distribution of burdens and benefits amongst research participants [58,60].

The same principles are stated in The Belmont Report, a document created in the USA in 1978 by the National Commission for the Protection of Human Subjects of Biomedical and Behavioral Research. This is a critical document for those involved in basic and clinical research with human beings [61].

Regarding clinical research, the regulations and mechanisms mentioned above seem to be effective in solving most of the ethical challenges [62], but that is not the case with invasive animal experiments, particularly those using NHPs [63,64]. The main reason for these inconsistencies seems to be the use of different frameworks to evaluate and guide research with humans and NHPs [65]. Guidelines and legislation that regulate human research rely on mostly deontological principles, while those that regulate animal research rely on utilitarianism. 

The use of different ethical frameworks for humans and NHPs may even result in opposing ethical recommendations: genetic experiments, which are often restricted from an ethical point of view when it comes to human beings [59,66], may be encouraged from an ethical point of view when it comes to NHPs [6,67].

In the next sections, we briefly describe both ethical frameworks and analyse how each applies to current NHP research. 

### 3.1. Deontology

Deontological ethics is the normative ethical position often associated with the philosopher Immanuel Kant, which judges the morality of an action based on the action's adherence to a rule or rules [68,69]. The underlying assumption is that something is good because it is the right thing to do [70]. Deontology stands for principles that must be fulfilled regardless of their consequences [71] and, according to Kant, there are hypothetical imperatives, which apply to someone who wishes to achieve a certain goal, and categorical imperatives, which are universal, absolute, and unconditional requirements that must be obeyed in all circumstances. Subsequent deontological philosophers, such as Ross [72], included the concept of moral relativism in deontology, which states that the morally right act is relative to the circumstances. 

Ross [72] also postulated seven prima facie duties: (1) duty of beneficence (help other people to increase their pleasure, improve their character, and so on); (2) duty of non-maleficence (avoid harming other people); (3) duty of justice (to ensure people get what they deserve); (4) duty of self-improvement (to improve oneself); (5) duty of reparation (to repay someone for acting wrongly towards them); (6) duty of gratitude (to benefit people who have benefited oneself); (7) duty of promise-keeping (to act according to explicit and implicit promises, including the implicit promise to tell the truth). It is noteworthy that current legislation and guidelines on research with human subjects encompass the first three. 

Deontology was established as an anthropocentric and rationalist framework due to the firm belief of rationalist philosophers that humankind is separated from the rest of animal kingdom by an exclusive capacity of reasoning. Since we now know that reasoning is not an exclusively human capacity, there is no reason why deontology should not be applied to non-human animals, as proposed by the American philosopher Tom Regan [73]. His theory of animal rights [73] asserts that every individual who is the subject of a life has inherent value. Such an animal is worthy of moral consideration, regardless of his/her species. According to Regan [73], individuals that fulfil the following criteria are “subjects of a life”: those who have beliefs and desires, perception, memory, and a sense of future, including their own future, an emotional life together with feelings of pleasure and pain, preference and welfare interests, the ability to initiate an action in pursuit of a goal, a psychophysical identity over time; and an individual welfare in the sense that their experiential life fares well or ill for them. According to Regan [73], “subjects of a life” ought to be respected and must not be treated as means to an end. 

#### Deontology in Contemporary NHP Research

Within biomedical research, NHPs are usually seen as merely means to an end. For example, xenotransplantation research aims for engineered animals lacking certain antigens so that their organs can be used for transplantation into human patients, with a reduced chance of immune rejection [8]. In such cases, the animal is being used as a means to an end. Although the same institutions (e.g., CIOMS) that wrote regulations and legislations to conduct clinical research also did so for animal research [74], the deontological principles that guided the former are totally absent in the latter. Nevertheless, European Directive 2010/63/EU on the protection of animals used for scientific purposes states that “the performance of procedures that result in severe pain, suffering or distress, which is likely to be long-lasting and cannot be ameliorated, should be prohibited” [75]. This incorporates, to some extent, the non-maleficence principle.

Even though the beneficence and non-maleficence principles can be found―to a certain extent―in some NHP laboratory research, the principle of justice is totally absent. As for the principle of autonomy, this can be found occasionally in cognitive research projects, when the test apparatus is built or presented in a way that animals enrol in the experiment of their own volition [41].

Several authors have recommended that NHPs (amongst other animals) involved in biomedical research should receive ethical consideration similar to that granted to humans, as well as analogous protection [46,60,76,77]. Most deontological guidelines require that the participant give informed consent prior to participation in research [59], but there are humans who cannot give valid informed consent (e.g., children or mentally incompetent adult patients). In such cases, the same deontological principles apply to research conducted on them, resorting to legal guardians, and specific legislation with greater restrictions. In most countries, humans who cannot consent may only be engaged in research that benefits them directly [78,79]. 

According to the legislation in most countries, research protocols for human studies―especially for humans who cannot provide consent―must be approved by independent experts (e.g., paediatricians in the case of children). Similar criteria could be used for NHPs who cannot consent, but who can effectively communicate their wishes through behavioural traits interpretable by an experienced primatologist. Additionally, if NHPs who are enrolled in a research experience had legal guardians, whose consent was mandatory prior to commencing research―as occurs with humans unable to understand or communicate informed consent [60,77], and as occurs in studies involving owners together with their companion animals [80]―then we believe that research involving NHPs would likely become more transparent and less exploitative than has sometimes reportedly been the case [81].

In sum, within research involving human subjects unable to consent, it is usually mandatory to have consent from (a) a legal guardian and (b) an expert on the condition that makes the human unable to consent. In NHP research, both conditions are usually absent: Not only there are no legal guardians whose task is to safeguard each NHP’s individual interests, but independent experts in primate behaviour do not normally verify protocol suitability for each animal.

It is interesting to note that it is largely amongst primatologists interested in studying NHPs by themselves, and not as models for humans, that we find the use of deontological principles and guidelines―mostly the beneficence and non-maleficence principles. For example, guidelines on darting arboreal primates state that darting cannot occur if the animal is facing the shooter, since the chest, face, neck, shoulder, thorax and lumber region, head or abdomen are unsuitable target sites that might harm the animals [82,83]. Similarly, semi-arboreal NHPs can only be darted on the ground [84]. These might exclude some animals from the sample in the same way some human participants are excluded from biomedical research, if they are at significantly increased risk of being harmed from an intervention or procedure [57].

### 3.2. Utilitarianism

Utilitarianism is a consequentialist ethical position which asserts that the value of an action is determined by the utility of its consequences, i.e., the morally right act is the one whose consequences maximise some form of utility (e.g., pleasure, wealth, wellbeing), for the majority [85]. However, the magnitude of pleasure and pain for all affected should receive equal consideration [86,87]. For example, it is morally justified to donate blood, because even though the number of individuals harmed is greater than the number of individuals who benefit from this action, it is a small harm, offset by a major gain. 

The most popular utilitarian views maintain that all sentient beings are moral subjects and their interests should receive equal consideration when deciding what is the morally right act [87]. NHPs are, beyond doubt, sentient beings. Hence, when using a utilitarian framework to evaluate the ethics of a biomedical procedure, the interests of NHPs that will be used as research subjects must receive the same consideration as the interests of those human beings who will benefit from the procedure. As a consequence, if the procedure is likely to cause serious harm (e.g., the death of NHP subjects) without bringing a substantial good (e.g., saving a greater number of human lives), it should not be conducted. 

Importantly, the ethical rules and laws that guide animal experimentation rely heavily on utilitarianism [59,81,88]. Most legislation on the protection of animals used for scientific purposes―including the current European Directive 2010/63/EU [75] states that the potential benefit of each research project should be balanced against the likely harm inflicted on the animals.

In many cases, the funding agencies evaluate potential benefits, while animal care committees review proposals in terms of animal harms. These committees do not directly interact, arguably impeding efforts to compare potential harms and benefits.

Even when this is not the case, the weighting scale is often misused either in predicting the benefits of the experiments, or in calculating expected harms. Below, we provide evidence of this, as it is virtually impossible to accurately predict the benefits, and in the calculation of harms, many variables are commonly left aside.

#### 3.2.1. Predicting Benefits from a Utilitarian Standpoint

NHPs are frequently used in drug trials which are often considered very promising [6]. However, retrospective examinations have demonstrated that the majority of those promising trials failed to translate to humans, or to produce the expected benefits [11], usually because of failures of safety or efficacy [89]. In fact, data from the Food and Drug Administration showed that 92% of drugs that succeed in preclinical tests fail to achieve their purpose within human clinical trials, and never reach the market [90]. These data were previously published in 2004, but more recent papers on the subject have demonstrated that there has not been a significant improvement: the success rate reportedly varies from 0.4% (in Alzheimer’s trials [91]) to 20% [92]. 

One of us (Andrew Knight) has systematically evaluated the contribution of chimpanzee research to biomedical progress, showing that approximately half of all publications describing chimpanzee research identified in a large-scale study were never cited by any subsequent paper, in any field, thereby making little obvious contribution to the ongoing advancement of knowledge. Even those chimpanzee studies that were cited by subsequent medical literature rarely made significant contributions to the development of therapeutic methods with significant potential for aiding human patients [9]. Bailey [10] also evaluated the role of chimpanzees in AIDS vaccine research, concluding that claims that chimpanzees have played a critical role in basic understanding of HIV-1 [93] were overstated.

More recently, Bailey and Taylor evaluated the contribution of NHPs to neuroscience research, demonstrating that there is a lack of robust evidence to support claims that NHPs are relevant and beneficial to human medical progress. These authors also concluded that human research methods, like functional magnetic resonance imaging with electrocorticography, are being simultaneously used in humans and NHPs for the same purpose, which, in their opinion, makes these NHP studies redundant [12]. Garner [89], on the other hand, maintains that the reason animal studies produce such poor results is the way they are performed, instead of the limitations of animal models.

The fact that one can frequently only evaluate the benefits achieved retrospectively markedly limits the suitability of utilitarianism in assisting an ethics committee to make an informed decision about whether a procedure should be permitted.

It is also important to mention the use of NHPs in experiments that will never reach human trials, due to ethical and legal limitations. Such is the case for experiments using NHP embryos or cloning experiments, which use NHPs due to their similarities with humans, although the use of humans in such experiments would be strictly forbidden [94]. Hence, potential benefits for human patients are absent or severely limited.

When predicting the potential benefits of a biomedical research project, all of humanity is usually considered to be potential beneficiaries. However, that is rarely if ever the case. According to the World Health Organization, approximately one-third of the people living in developing countries are unable to receive or purchase essential medicines on a regular basis [95].

Finally, all basic research produces knowledge, which is in itself a benefit to humankind, since scientific knowledge has cultural value in itself. Nonetheless, this benefit is hard to quantify, or to balance against concrete and substantial costs.

#### 3.2.2. Assessing and Predicting Harms from a Utilitarian Standpoint

While addressing the harms inflicted on non-human animals, including NHPs, researchers tend to focus on the severity of the procedures described in the experimental protocols and overlook other harms. In fact, European Directive 2010/63/UE, with the aim of regulating the level of severity inflicted on laboratory animals, includes an annex on the severity classification of procedures [75]. This may reinforce the propensity to disregard other sources of pain and distress.

Unlike humans, NHPs cannot be informed about their procedures―hence, even a painless procedure like an MRI can be terrifying for a naïve NHP [46,76]. To always classify this procedure as “mild”, in accordance with current European legislation, ignores subjective experiences, such as fear, that might vary individually.

Wild-caught NHPs also experience anxiety and pain during capture, in holding facilities, and often lengthy transportation and confinement, whereas laboratory-bred NHPs may undergo suffering during breeding, and from maternal separation, potentially much earlier than would occur in the wild [96,97]. It is noteworthy to mention that due to the intense stress caused by wild capture, the UK banned the wild capture of primates for their use in research in 1996. Similarly, European Directive 2010/63/EU states that only the offspring of wild-caught NHPs can be used in research experiments.

NHPs who live under laboratory confinement conditions may experience pain and distress not only during procedures, but also during many other situations that are not normally considered when evaluating the harms and benefits of the research. Self-injurious behaviour is an obvious sign of stress that has been extensively described in NHPs living in laboratories (for a review, see Reference [98]). Similarly, floating limb syndrome, which can be defined as raising the arms or legs without an obvious function, is a readily identifiable stress-related behaviour [99]. Another easily recognisable sign of stress in NHPs is the freezing response. In both humans and NHPs, this response is a common and immediate response to threat situations that allows the individual to evaluate the danger and decide how to deal with it [100]. When there is a dysregulation in fear response (e.g., post-traumatic stress disorder—PTSD), this behaviour may emerge in non-threatening or mildly threatening situations and may last for prolonged periods [100]. Hence, inappropriate freezing behaviour is a signal of fear and anxiety that researchers should not ignore, regardless of the stimulus. Some types of behaviour, such as a high frequency of self-grooming, are stereotypic abnormal behaviours in some species but not in others [100]. However, checking species-specific ethograms and normal activity time budgets could help to identify such abnormal behaviours.

Species ethograms can also be helpful in identifying the naturally occurring behavioural repertoire. In laboratory housing, most NHPs face restrictions on performing certain natural behaviour patterns. This is not usually considered when assessing harms. The same may occur when an NHP witnesses the harming or killing of peers [96]. Additionally, experiencing stress, especially at an early age, impacts the NHP immune system and brain structures [101,102]. These long-term stress-related harms are not normally considered when assessing animal welfare impacts and might even reduce the suitability of some NHPs as models for humans [101].

Facial expressions could be an important tool to understand NHP emotional states, since they often convey emotion or pain in many different NHP species (for reviews, see References [32,103,104]). In the case of chimpanzees, for example, the expression of a full closed grin as described by Goodall [27] is reliably associated with fear, distress, and painful contexts [32,103]. Similarly, in rhesus monkeys, a grin signals fear or submission [26]. In recent years, the facial action coding system (FACS) developed by Ekman and Friesen [105] has been adapted to several NHP species, like chimpanzees [106], rhesus monkeys [107], gibbons [108], and orangutans [109]. This tool could help researchers to more objectively assess NHP emotions.

With the help of such tools, it would become easier to evaluate which procedures should be prohibited or modified in order to spare NHPs from severe pain or stress. Their use has been suggested for NHPs [46,61], but has not yet been widely implemented [81].

## 4. Ethical limitations of 3Rs Principles 

Current policies underpinning animal experimentation follow the 3Rs principles, first described by Russell and Burch [110]. These principles assert that whenever possible, animal models should be replaced with alternative methods; the number of animals used in experiments should be reduced to a minimum; and their suffering should, whenever possible, be ameliorated, e.g., through humane endpoints, less invasive procedures, and the use of anaesthesia (refinement).

Replacement is the first and, in our view, the most important of the 3Rs. Its achievement in a particular case makes implementation of additional Rs unnecessary. However, replacement is often grounded in the unverified assumption that animals are good models for human diseases―an assumption that is increasingly challenged by empirical evidence (for a review, see [111]). To gain regulatory acceptance and/or be funded, alternative methods often need to demonstrate that they can provide equivalent or superior data to those obtained through animal testing, even when the current animal model results are variable rather than consistent, and even when these models have often failed to reliably predict human responses to drugs [112]. This *status quo* approach delays the development of promising non-animal methods in toxicity and drug testing and diverts biomedical research away from non-animal methods.

These 3Rs principles underpin virtually all legislation and guidelines concerning the use of animals in scientific procedures. However, they do not offer a philosophically consistent ethical framework and are insufficient to address ethical concerns regarding NHP use within biomedical research.

The 3R policies comply―to some extent―with utilitarianism, since reduction and refinement are tools used to try to minimise the total amount of harm inflicted. However, they do not provide tools to predict benefits, or the extent of long-term harm, which makes them insufficient to fulfil the requirements of utilitarian analysis.

Whilst public health advancement might be a justifiable goal, from a utilitarian standpoint, the pursuit of biomedical NHP research (that might provide only modest benefit) might not be justifiable. From a deontological point of view, the 3Rs are largely irrelevant, since they do not prevent the research subjects from being used as means to an end. Additionally, the 3Rs do not comply with principles of autonomy or justice, which are crucial within the deontological approach prescribed by Beauchamp and Childress [58].

## 5. Societal Determination of Ethical Frameworks

Whenever animal-based research is the topic of discussion, the balance between competing perspectives is often decided at the societal level, and the prevailing culture enables or proscribes a certain type of scientific activity [113].

When it comes to science, people tend to support animal experiments according to utilitarian principles, i.e., people consider the potential benefits for humanity when assessing their level of support for certain research [15,48,113,114,115]. However, when it comes to animals that people consider companions, such as domesticated dogs, the number of people who support their use in scientific research decreases dramatically, regardless of the perceived potential benefits for humankind [48]. With these animals, people shift their ethical paradigm, applying the beneficence and non-maleficence principles.

The emergence of ethical decisions is influenced by the feeling of discomfort that most people experience when confronted with the suffering of others, and their own sense of wellbeing and fulfilment when contributing to the alleviation of pain or the promotion of happiness [116,117]. However, these decisions rely on available information about the phenomenological experiences of others. Closeness, familiarity, and knowledge of animals, including NHPs, have all been variables linked to increased empathy for animals [118] and less tolerance for animal use in invasive or harmful scientific research [113].

People are more willing to accept research on nonhuman animals, including NHPs, if they believe animals are comfortable and well cared for, and in the mid-20th century, according to the National Opinion Research Centre, 75% of the public believed that medical schools treated laboratory animals as well as individual owners would [114]. Most owners consider their pets as individuals with intrinsic value, and veterinary clinical research conducted on pet dogs follows deontological principles similar to the ones used in human clinical research [119]. 

The way animal husbandry is portrayed, as well as the level of familiarity people have with different species, are thus critical features of engaging society with either utilitarian or deontological ethical frameworks. Accurate portrayal of the actual state of both variables, as well as a realistic portrayal of human healthcare benefits that arise from animal research, would, in our opinion, lead to stronger support for application of the deontological framework.

## 6. Ethical Research with NHPs

There is no robust evidence that we need NHPs to model specific human diseases [9,10,11]; therefore, there is no overwhelming moral or scientific reason to confine NHPs within laboratories, to be invasively used as defective models for human disorders. In fact, Garner and colleagues recently [89,120] suggested that in order for biomedical research using non-human animals to be more effective, they should be treated as patients. We agree with this view but emphasise that this is not possible for animals confined in a laboratory.

### 6.1. Ethical Research with Possible Healthcare Applications

Disorders that affect humans and NHPs should ideally be studied using NHPs who suffer naturally from the disorder concerned, either in wild populations, or in captive NHPs who need treatment.

In 1966, Jane Goodall witnessed a polio outbreak in wild chimpanzees living at Gombe Stream National Park (Tanzania). In some individuals, the subsequent disability was so severe that some animals were euthanized [121]. Instead of infecting healthy laboratory animals with polio, these wild chimpanzees who succumbed to polio from natural causes could theoretically have been studied to understand polio. The knowledge acquired from these studies would have been useful for science in general, and for infected chimpanzees specifically―hence upholding the justice principle. It might or might not have been useful for humans but, given the more natural induction and progression of the disease, it could have been more useful than similar research performed on laboratory chimpanzees. Although the laboratory environment allows for the control of possibly confounding variables, and manipulation of the exact time of infection, this level of control and information is rarely possible with human patients. Wild animals that naturally acquire a disease occurring in humans and other species can be a better model than laboratory animals, since―just like human patients―they are living in a complex environment where social and natural variables can modulate disease progression. In human patients, it is very hard or even impossible to determine the exact time of infection and what other variables (e.g., inadvertent exposure to external viruses) could interfere with disease progression and/or clinical trial results. Even researchers that support the use of animals as models for human disorders acknowledge that standardisation of too many variables in the laboratory can be a limitation, rather than a strength [89]. 

There are NHPs previously used by industries (e.g., entertainment, biomedical research) and subsequently suffering from psychological and behavioural disorders, for whom psychiatric/psychological treatment is not only appropriate, but also a moral imperative [122,123,124]. Using these animals as research patients for PTSD, for example, could benefit both science and these particular animals. Again, the data obtained might or might not be useful for human healthcare, but the results obtained from laboratory animals would not necessarily be more useful.

Epidemiological studies with wild populations can also be conducted with minimal disturbance of the animals [125,126], hence respecting the autonomy principle.

### 6.2. Basic Ethical Research

Some may argue that NHP research facilities are useful for purposes other than medical research. This is the case for basic research, which, according to the Frascati Manual [127], is “experimental or theoretical work undertaken primarily to acquire new knowledge of the underlying foundation of phenomena and observable facts, without any particular application or use in view” [127]. The majority of such basic research is laboratory-based.

However, a large amount of such research can be conducted in non-invasive field studies, using wild populations―hence respecting the individuals as subjects with inherent value. For example, Kano and colleagues used a laboratory apparatus to study differences in gaze behaviour in chimpanzees and bonobos, concluding that bonobos pay more attention to the eyes and face of other individuals [128]. On the other hand, Fröhlich and colleagues reached the exact same conclusion by observing communicative interactions in mother-infant dyads of wild populations [129]. Similarly, Fujita and colleagues created an experimental laboratory procedure to study capuchin monkeys’ deceptive behaviours [130], while others [31] were able to study tactical deception of the same species in the wild, gathering more robust and reliable data on the subject. Another good example which has been widely studied is chimpanzee communication: while in captivity, only 31 gestures were described [131], observations in the wild raised this number to 66 distinct gesture types to communicate at least 19 different messages [33]. In fact, most of what we know about behaviour and the ecology of NHPs has come from long-term field studies [17,27,132,133,134], and recent field studies continue to amaze us by revealing new species [135], as well as unexpected behaviour from well-known species, such as bonobo hunting [136], and some behaviours that might be ritual practices amongst wild chimpanzees [137,138].

These studies have been conducted respecting the beneficence and non-maleficence principles, and even the principle of justice, since the knowledge obtained from those data, some of it also presented as documentaries and in other forms for widespread media dissemination, raises awareness of animal emotion and cognition [139], potentially increasing empathy towards these NHPs, and ultimately increasing the impetus for their conservation.

However, there are research questions, either in safety and efficacy testing or fundamental research, where it is not possible to obtain knowledge using only observational techniques (e.g., genetics, neuroscience). However, there are ways of continuing this research without overlooking ethical constraints.

In questionable situations, it is always pertinent to ask whether the knowledge acquired through the suffering of other animals complies with the bioethical principles described by Beauchamp and Childress [58]. In the literature, we can find examples of basic research with non-human animals that fulfil these principles. Berns and colleagues [140] used positive reinforcement to train dogs to stay still within a functional MRI device. The dogs were unrestrained and free to leave the device at all times, including during training sessions (autonomy principle). Without harm or distress, much fundamental knowledge on the canine brain was obtained (non-maleficence principle), which may ultimately benefit the wider canine population (principle of justice). The researchers would gradually play louder sounds in the surrounding environment so that animals would not get startled by MRI sounds (principle of non-maleficence). This innovative method has been providing exciting insights into the canine brain [141,142,143], and a similar technique could be used to study NHPs living under semi-natural conditions, replacing neuroscience NHP laboratories where even the least invasive techniques [144] require temporarily restraining fearful animals. Some NHPs share habitat with human beings (e.g., rhesus monkeys in India or Nepal) and sometimes even enter and explore human homes, which should make it possible to conduct experiments with these animals similar to those described above with dogs. Some NHPs species are particularly harmless and cooperative (e.g., marmosets or capuchin monkeys). Individuals from those species who are held captive for other reasons (e.g., rescued animals living in sanctuaries) could also be enrolled in experiments similar to the ones conducted by Bern and colleagues on dogs [140,141,142,143]. Even potentially dangerous NHPs, such as chimpanzees, can participate in experiments consensually, in the same way human participants do [41].

## 7. Conclusions

In light of the current knowledge, the use of NHPs in basic research warrants something of a paradigm shift. We propose that basic research with NHPs should continue only if carried out under the same ethical deontological criteria that guide basic research with human beings. 

Whenever non-invasive basic research protocols require the use of NHPs, the participants should be recruited from sanctuaries or similar facilities. Local legal guardians of NHPs should evaluate the procedures to verify whether the principles of autonomy, beneficence, non-maleficence and justice, as defined by Beauchamp and Childress [58], have been fully incorporated. That being the case, the legal guardian would provide the necessary informed consent.

By complying with such standards, we would not only grant other primates a level of respect and protection consistent with that we provide to members of our own species, but we would also be encouraging researchers to develop better research protocols and higher standards for captive management, which could, in turn, result in improvements in data quality, and in the reliability of some research results.
